# The Influence of Erotic Stimulation on Brand Preference of Male and Female Consumers: From the Perspective of Human Reproductive Motives

**DOI:** 10.3389/fpsyg.2022.848864

**Published:** 2022-06-07

**Authors:** Xia Wei, Xin Huang, Yufeng Xie, Rungting Tu

**Affiliations:** ^1^College of Management, Shenzhen University, Shenzhen, China; ^2^Faculty of Economics and Business Administration, Sapporo Gakuin University, Ebetsu, Japan

**Keywords:** erotic stimulation, mate-attraction motive, mate-retention motive, brand personality, gender difference, consumer perception, cognitive projection

## Abstract

Based on the theory of human reproductive motives, this study discusses how erotic stimulation can help activate male and female consumers’ reproductive motives (i.e., mate-attraction and mate-retention motives), influencing product purchase intention and brand personality perception. Specifically, the results of four experiments reveal that when a man receives erotic stimulation from women, his mate-attraction motive is activated. Consequently, he perceives that the brand personality of his possessions is more rugged, and his preference for products with a rugged brand personality is stronger. Unlike male consumers, when a heterosexual woman in an intimate romantic relationship receives erotic stimulation from other women, her mate-retention motive is activated. As a result, she perceives that the brand personality of her partner’s possessions is sincerer and prefers to purchase products with a sincere brand personality for her partner. This effect will not occur when a product is owned by her male colleague than her partner.

## Introduction

Erotic stimulation, essentially as a kind of sensory cue, is mainly visual such as nudity and innuendos, so it is frequently used in advertising strategies to attract consumers’ attention (Reichert et al., 1999, 2001; Reichert and Lambiase, 2003; Reichert and Carpenter, 2004). Sometimes even when products are not directly related to these sexy elements, they still put erotic stimulation into advertisements (Lambiase and Reichert, 2013). The most common sexy advertisements target male consumers and show female sex appeal (Saad, 2004; Reichert et al., 2012). Many branding companies hope to utilize sexy elements in advertising to give consumers strong visual stimulation and deep impressions, and establish a positive connection between their products and sexy spokespersons (Dahl et al., 2009; Griskevicius and Kenrick, 2013; Hornik et al., 2017).

However, it remains controversial whether erotic stimulation in advertisements can bring real benefits to brands. Sengupta and Dahl (2008) found that women did not like advertisements with erotic stimulation unless they had an open mind about sexuality. Many male consumers could not describe product details after watching sexy advertisements of products (Samson, 2018). Moreover, many studies have focused on the impact of sexy advertisements on individuals’ self-related decisions, while neglecting partner-related shopping decisions. For example, men receiving erotic stimulation prefer to purchase luxury and conspicuous products for themselves (Griskevicius et al., 2007; Sundie et al., 2011; Gurzki and Woisetschläger, 2017; Bradshaw et al., 2020). Women are prone to buy conspicuous products for themselves to show ownership (Wang and Griskevicius, 2014; Zhao et al., 2017). Although the scenario of women shopping for men is prevalent in reality, little is known about how sexy advertisements designed to appeal to male consumers influence the purchasing decisions of heterosexual women for their partners.

Therefore, to explore the issue, we first introduce the reproduction theory. Because women are usually concerned about their appearance (Roux et al., 2017), they are sensitive to the threat of other beautiful women (Maner et al., 2012; Li and Meltzer, 2015) and are inclined to stay away from the threat (Lydon et al., 2003; Krems et al., 2017) to maintain romantic relationships with partners to facilitate reproduction. Thus, we think that advertisements with sexy women make female customers feel their intimate relationships threatened, resulting in mate-retention motives. On the other hand, magazine advertisements with photos of young women trigger men’s mate-attraction motives (Bleske-Rechek and Buss, 2006). Next, according to cognitive projection theory, motives can affect an individual’s self-perception and perception of others (Fiske, 1992; Haselton and Buss, 2000; Lemay et al., 2007, 2010). Hence, we argue that when a woman’s mate-retention motive is triggered, she believes that her partner is also willing to maintain the intimate relationship and is sincere. In contrast, mate-attraction motives may make men more rugged. Finally, based on person-brand relationship theory, a person’s belongings can reflect the person’s personality to some extent (Belk, 1988; Misra and Beatty, 1990; Ahuvia, 2005; Cunningham et al., 2008; Malär et al., 2011; Borau and Bonnefon, 2020; Wang et al., 2022). Thus, we consider that after receiving erotic stimulation from other women, a woman in an intimate relationship woman thinks that her partner’s possessions, such as a leather bag and a car, have the same personality as the owner and are sincere. Nevertheless, men perceive that their belongings have a rugged brand personality conducive to attracting the opposite sex. In what follows, we establish the connection between concepts by reviewing the relevant literature, and further formulate research hypotheses. We then find men’s and women’s preferences for the ideal male personality under two motives through a pre-experiment, and further test the hypotheses through two sets of experiments. Finally, we discuss the findings, possible contributions, and future research directions.

## Conceptualization and Hypotheses

### Two Motives for Reproduction

Erotic stimulation causes sexual associations and thus directly rouses sexual motivation. Reproduction is regarded as an inherent and fundamental need in human evolution, used to explain individual responses related to gender relations. There are two basic human motives that facilitate reproduction: the mate-attraction motive and the mate-retention motive (Buss and Shackelford, 1997; Maner et al., 2007). Erotic stimulation from women triggers heterosexual men’s motive to attract female partners (Bleske-Rechek and Buss, 2006). Meanwhile, erotic stimulation from other women impels heterosexual women in intimate relationships to focus on sexual attraction and potential threats to intimacy, which triggers a mate-retention motive in intimate relationships.

**The mate-attraction motive** aims to obtain a mate with a potential probability of reproduction in a short time, and its key point is to increase individual attraction to the opposite sex. Individuals judge and select potential and actual partners according to four primary dimensions: warmth-trustworthiness, vitality-attractiveness, status-resources, and confidence-humor (Gerlach et al., 2019). In particular, for males, status attainment and promotion contribute to unique motives for courtship (Krems et al., 2017). Therefore, the mate-attraction motive makes men more manly (Baumeister and Vohs, 2004), more creative (Griskevicius et al., 2006), more willing to take risks (Bakerjr and Maner, 2008), healthier, more energetic (Vandenbroele et al., 2020), more independent (Griskevicius et al., 2006), more heroic (Griskevicius et al., 2007), and more distinctive (Griskevicius et al., 2007). In addition, males are more willing to help others in situations where they can exhibit heroism and dominance than in general situations (Sundie et al., 2011). From the perspective of consumption, the mate-attraction motive makes consumers pay more attention to products showing their status (Janssens et al., 2011) and prefer to purchase luxury products (Griskevicius et al., 2007; Bradshaw et al., 2020) and conspicuous brands (Sundie et al., 2011; Gurzki and Woisetschläger, 2017). However, the mate-attraction motive makes women more caring (Griskevicius et al., 2007) and more willing to show their beauty (Kenrick and Keefe, 1992; Hill and Durante, 2011) and cooperation (Griskevicius et al., 2006). Accordingly, under the influence of mate-attraction motives, both men and women tend to become more distinctive and outstanding (Berger and Shiv, 2011). Although different people use different strategies to attract the attention of the opposite sex, their behaviors are motivated essentially by the mate-attraction motive, so these behaviors are usually visible, demonstrative, and emblematic (Griskevicius et al., 2007; Otterbring et al., 2018). It can be seen that when men are motivated by mate attraction, they will act more rugged rather than sincere.

However, there are significant differences in the attitudes of men and women toward sex. For instance, men tend to see the acquisition of sex as a goal, while women are more inclined to regard sex as a commitment to an intimate relationship (Dahl et al., 2009). Unlike men, women view sexual engagement as a commitment to maintaining intimacy and associate sex with long-term relationships. Especially for these women in an intimate relationship, it is easy to generate a mate-retention motive when the relationship is potentially threatened.

**The mate-retention motive** aims to maintain a long-term relationship with a specific mate. This motive requires individuals to actively maintain existing relationships and eliminate potential relationship threats to prevent relationship breakdowns and loss of reproduction opportunities (Redlick and Vangelisti, 2018). For example, individuals maintaining intimate relationships usually act more caring toward their partners (Saad and Gill, 2003) and give lower appraisals to other potential relationship threats (Lydon et al., 2003). However, compared with mate attraction, mate maintenance often means that individuals need to invest more time, energy, money, and other resources (Griskevicius and Kenrick, 2013), and most of the strategies employed are not visible and demonstrative.

Although these two motives are both related to reproduction, they have different modes of operation and may lead to completely different cognitive and behavioral responses. There are differences in the activation methods for the two motives. The mate-attraction motive is usually stimulated by real or imaginary potential partners, while the mate-retention motive can be actuated by potential spoilers or relationship threats (Griskevicius and Kenrick, 2013). Specifically, the same erotic stimulation from another woman means the existence of a potential partner for a man, while for a woman in an intimate relationship, it may mean the existence of a potential rival. Some researchers have pointed out that erotic stimulation from women can activate men’s mate-attraction motives (Bleske-Rechek and Buss, 2006). Furthermore, the mate-attraction motive tends to direct an individual’s attention to the opposite sex, regarded as a potential target of mate attraction (Maner et al., 2005). On the other hand, women are more concerned about appearance in intimate relationships and are more sensitive to the threat of competitors in terms of appearance. For instance, some scholars have found that women are more sensitive to the opinions of others and tend to make a good impression through appearance (Roux et al., 2017). In addition, women tend to allocate more attention to beautiful female faces (Li and Meltzer, 2015) and give lower appraisals to the people with such potential relationship threats (Lydon et al., 2003). They are more inclined to stay away from women ovulating (Krems et al., 2017) and are more prone to use conspicuous products to send possessive signals to other persons perceived as potential relationship threats (Wang and Griskevicius, 2014; Zhao et al., 2017). Therefore, when a woman in an intimate relationship receives erotic stimulation from other women, she is easily motivated to maintain the existing intimate relationship. When the mate-retention motive is activated, individuals’ attention is more likely to be drawn to the highly attractive same-sex representatives (Maner et al., 2007). Thus, the mate-retention motive often directs an individual’s attention to the same-sex viewed as a potential threat to existing intimate relationships (Maner et al., 2009), and women are more inclined to classify them based on facial attractiveness (Maner et al., 2012).

### Cognitive Projection of Motives

The powerful role of motives has been explored in sociology, psychology, and marketing. Some studies have shown that motives can affect not only an individual’s way of thinking and acting but also the individual’s perception of others (Fiske, 1992; Haselton and Buss, 2000). For example, when individuals would like to build relationships with attractive persons, they tend to think that these good-looking people are easy to approach (Lemay et al., 2010); when individuals care about their partners, they believe that their partners also care about them (Lemay et al., 2007); when individuals are ostracized by society and avoid further interpersonal threats, they think others are more hostile (DeWall et al., 2009). Thus, a woman in an intimate relationship feels a threat to the relationship after receiving erotic stimulation from other women, and the mate-retention motive is likely to be projected onto her intimate partner.

Individuals’ interpersonal goals may affect their cognition of the target population, thereby producing cognitive biases. For example, the belief that goals are easier to achieve is a positive functional cognitive strategy. Similarly, if individuals in interpersonal relationships are motivated to maintain relationships, they are likely to have certain perceptual biases and even unrealistically optimistic attitudes toward the other person involved (Simpson et al., 1995; Murray et al., 1996; Rusbult et al., 2000; Lemay et al., 2007; Lemay and Neal, 2013). Therefore, when the mate-retention motive is activated, an individual may think that the partner also owns motivation and characteristics and makes efforts to maintain the relationship.

In the field of consumption, the perception of personal motivation and characteristics can be reflected in the items they own. Meanwhile, the effects of motivated projection can be reflected in the partner and partner’s possessions. The items owned by an individual are usually regarded as an extension of the individual and a part of them, helping people complete their self-recognition and identity construction (Belk, 1988; Ahuvia, 2005; Cunningham et al., 2008; Borau and Bonnefon, 2020; Wang et al., 2022) based on a widespread belief, “You are what you buy.” We think that the mate-retention motive is triggered when a woman in an intimate relationship receives erotic stimulation from other women, and the motive may be projected onto her partner’s possessions. Moreover, the woman is willing to perceive the brand personality of her partner’s possessions as more conducive to maintaining the intimate relationship.

### Product Owners and Brand Personality

The information conveyed by owned products and brands is often related to the brand personality (Aaker, 1997; Johar et al., 2005). Brand personality endows the brand with human-like traits and plays an important role in understanding the relationship between the brand and consumers (Aaker et al., 2004). Consumers tend to use brand personality to show their true or ideal selves (Belk, 1988; Aaker, 1997, 1999). Because they believe that the owner’s personality is consistent with the brand personality (Misra and Beatty, 1990), they prefer brands that fit their self-concepts (Swaminathan et al., 2009). Similarly, people make value judgments about others based on their purchasing behavior (Baran et al., 1989).

However, consumers’ perception of a brand’s personality is not static, so any direct or indirect contact between consumers and the brand may affect their perception of the brand personality (Plummer, 1985). The owner of branded products also helps shape individuals’ perception of the brand, which is one of the essential roles of brand spokespersons. Because consumers are inclined to think that the owner’s personality is in line with the brand personality (Misra and Beatty, 1990), cognitive consistency can play a significant role in forming the brand personality perception.

According to the principle of owner consistency (Misra and Beatty, 1990; Malär et al., 2011), consumers’ perception of brand personality may be influenced by the perception of the owner’s personality. The effect of the mate-retention motive can be projected on partners, making individuals think that their partners are more willing and able to maintain the existing relationship than they actually are. Aaker (1997) constructed a basic model of brand personality and proposed the following five typical dimensions: sincerity, excitement, competence, sophistication, and ruggedness. Based on previous research results, brand personality related to erotic stimulation toward male consumers may be mainly reflected in two dimensions: ruggedness and sincerity. When sexually aroused, men pay more attention to sex itself (Dahl et al., 2009), so they want to be more masculine, and ruggedness is a typical masculine trait. Solomon (1983) found that adolescent males tend to use strongly “macho” products (cars, clothing, cologne, etc.) to enhance their fragile self-concept of masculinity. Some researchers also found that men prefer products associated with robust masculinity, such as off-road vehicles, when their sense of masculinity is threatened (Willer et al., 2013). Thus, when faced with the erotic stimulation from attractive women, men’s mate-attraction motives will be activated and thus enhance their rugged perception toward his possessions and prefer the products or brands with rugged personality.

From the perspective of relationship maintenance, previous studies have pointed out that ideal social relationships are often associated with sincerity. For example, socially excluded consumers prefer and feel a stronger connection with brands generally perceived as sincere (Min, 2012). When faced with erotic stimulation, men will reduce their preference for romance-related products representing long-term relationships (Brough et al., 2016), while women are more interested in the commitment to the relationship represented by sex and the intimate relationship itself. Because sincerity is a vital attribute to fostering good interpersonal relationships for women, they will sport sincere brands to present themselves as sincere persons in order to re-establish social connections and gain a sense of belonging (Dahl et al., 2009). Moreover, people who lack confidence in their worthiness being loved (i.e., high anxiety) but want to be accepted by others (i.e., low avoidance) are more likely to prefer sincere brands, and this preference for sincere brands is especially evident in situations of overt consumption with high interpersonal expectations (Swaminathan et al., 2009). As a result, women are also more likely to experience anxiety and lack of confidence when faced with erotic stimulation targeting their partners from other attractive women, and are more inclined to choose sincere brands for their partners.

Because motives modulate an individual’s perception of others and further affect the individual’s evaluation of the personality of the brands owned by others, we hypothesized that this evaluation could be influenced by the stimulation of different motives and formulated this in the following way:

**Hypothesis 1 (H1):** When a man receives erotic stimulation from women, (a) his mate-attraction motive is activated, (b) he perceives that the brand personality of his possessions is more rugged, (c) his preference for products with rugged brand personality is stronger.

**Hypothesis 2 (H2):** When a woman receives erotic stimulation from other women, (a) her mate-retention motive is activated, (b) she perceives that the brand personality of her partner’s possessions is sincerer, (c) she prefers to purchase products with a sincere brand personality for her partner.

**FIGURE 1:**
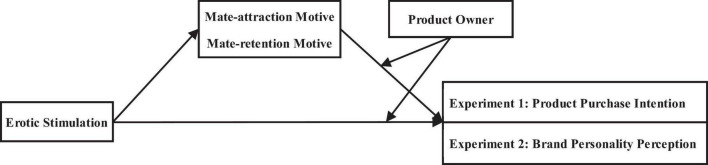
Theoretical framework.

We conducted two sets of experiments to test our hypotheses. Experiments 1a and 1b examined how erotic stimulation directed at men affects male costumers shopping for themselves and female costumers in intimate relationships shopping for partners. Experiments 2a and 2b revealed that relational motivations inspired by erotic stimulation are projected onto the brand personality of the product, which reflects the relationship the perceiving subject would like to establish with the owner of the product (see Figure 1 for the theoretical framework).

## Materials and Methods

### Experiment 1: The Effect of Erotic Stimulation on the Purchase Intention of Products With Different Brand Personalities

In Experiment 1, we conducted two separate sub-experiments, 1a and 1b, to examine how the same erotic stimulation affects male and female consumers’ preferences for brand personality when they purchase products.

#### Experiment 1a: The Effect of Erotic Stimulation on Men’s Preference for Brand Personality

##### Method

Experiment 1a tested whether erotic stimulation from sexy women increased male consumers’ preference for products with a rugged brand personality. In the experiment, we adopted a double-factor between-subjects design of 2 (erotic stimulation: yes vs. no) × 2 (brand personality: ruggedness vs. sincerity). Based on the typical effect sizes reported in the social psychological literature (Richard et al., 2003), a minimal meaningful effect size of *f* = 0.4 was selected. An *a priori* power analysis with 95% power indicated 115 participants as the minimal participant size needed. Our study was aimed at heterosexual groups, so non-heterosexual groups were excluded. A total of 143 participants (*M*_age_ = 29.61) were recruited and randomly assigned to one of the four groups. Each participant was given a questionnaire consisting of two separate surveys.

In the first part, we used pictures to manipulate erotic stimulation according to Li and Zhang (2014; see Supplementary Material). In the group receiving the erotic stimulation, participants were told that a famous car company was shooting a new advertisement for its new car; they were shown four pictures of sexy female car models and asked to answer some questions. We set up six questions and asked them to evaluate the characters in the four pictures for various aspects, including “Which model is the most beautiful?” “Which model has the best body?” and “Which model is the sexiest?” Then, participants were asked to answer the question, “If you were the director of brand marketing, which model would you choose to be the spokesperson of your brand?” Comparing four sexy models is only to enhance the authenticity of the scenario design and the effect of the erotic stimulation. In the control group, participants were told that a travel company was shooting a new print advertisement for travel planning; they were shown four scenery posters and asked to answer some questions. We also designed six questions to ask these participants to evaluate the scenery in the four posters for various aspects, including “Which poster has the most beautiful view?” and “Which poster is the most attractive?” The participants were then asked to answer the question, “If you were the director of brand marketing, which poster would you use for the advertising campaign?” According to Otterbring and Sela’s (2020) manipulation test for sexual stimuli, in both the erotic stimulation and scenery groups, all participants were asked to rate the pictures they saw (1 = strongly disagree; 7 = strongly agree) for three aspects, namely “these pictures are sexy,” “these pictures are hot,” and “these pictures are titillating.”

Bags are common belongings for men and reflect the taste of their owners, according to previous literature (Septianto et al., 2021). Thus, in the second part, we asked the male participants to buy a leather bag for themselves and showed them the same bag’s picture from a brand’s website, and then manipulated the brand personality with different advertising texts, as follows:

###### Sincerity

A leather bag can reflect its owner’s personality, so it is not only related to the owner’s taste but also affects others’ evaluation of its owner. Men’s understated attitude toward bags makes them fond of deep and natural colors suggestive of sincerity, and sincere men also have their views on the pursuit of famous brands. The men’s bag of the Gothic brand is sincere and unobtrusive. It has no flashy and cumbersome decorations, and its color is like a cup of pure and strong coffee, which is endlessly memorable. The exquisite workmanship reveals composure and concentration, showing men’s sincerity.

###### Ruggedness

A leather bag can reflect its owner’s personality, so it is not only related to the owner’s taste but also affects others’ evaluation of its owner. Men’s flamboyant attitude toward bags makes them fond of deep and natural colors full of ruggedness, and rugged men also have their views on the pursuit of famous brands. The men’s bag of the Gothic brand is rugged and masculine. It embodies the brand’s unique personality, including a rugged shape and textured crocodile bone spurs. It blends wildness and power, making it seductive. The unique design highlights the unbridled and untamed temperament, showing men’s ruggedness.

Then, we asked each group to rate the bag in a specific scenario (ruggedness or sincerity) using a 7-point scale to measure participants’ purchase intention. Finally, they answered additional items for further analysis: “Do you know the brand of the leather bag?” “How old are you?” and “Do you maintain a stable and intimate relationship with your girlfriend?” If the participants know the brand in advance, we will exclude it to avoid the impact on the experimental results. At the same time, we will control age and intimacy as covariates in further analysis.

##### Results

We kept 140 valid participants and removed the other three participants, including one participant who knew the brand of the leather bag and two participants who had many contradictions and omissions in their answers.

The erotic stimulation manipulation test revealed that participants in the erotic stimulation group (*M* = 6.43, *SD* = 0.56) rated the sexiness of the pictures significantly higher than those in the scenery group [*M* = 3.96, *SD* = 1.76, *t*(138) = 11.19, *p* < 0.001, η^2^ = 0.48]. The brand personality manipulation test showed that participants in the ruggedness group (*M* = 5.54, *SD* = 1.73) scored significantly higher on the perception of ruggedness than those in the sincerity group [*M* = 2.67, *SD* = 1.49, *t*(138) = 10.51, *p* < 0.001, η^2^ = 0.44]. Therefore, the manipulation of erotic stimulation and brand personality was successful.

**FIGURE 2:**
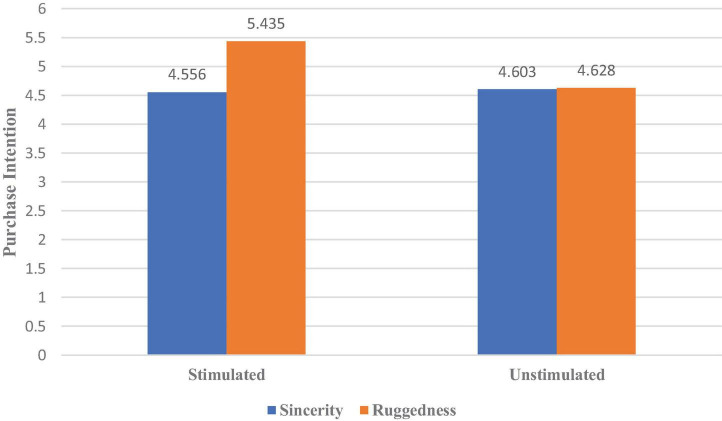
The effect of erotic stimulation on men’s preference for brand personality.

Next, we conducted an analysis of covariance with erotic stimulation and brand personality as independent variables, purchase intention (Cronbach’s α = 0.788) as a dependent variable, and age and whether the intimate romantic relationship was stable as control variables. The results revealed a significant interaction between erotic stimulation and brand personality [η^2^ = 0.063, *F*(1,136) = 10.39, *p* = 0.002] (Figure 2). The simple-effects analysis further revealed that erotic stimulation increased men’s preference for the bag exhibiting ruggedness [η^2^ = 0.124, *F*(1,136) = 19.023, *p* = 0.000] but did not affect men’s preference for the bag embodying sincerity [*F*(1,136) = 0.061, *p* = 0.806]. Therefore, H1c is supported.

#### Experiment 1b: The Effect of Erotic Stimulation on Women’s Preference for Brand Personality

##### Method

Experiment 1b examined whether erotic stimulation from other women increased female consumers’ preference for sincere brand personality when choosing products for their partners in stable relationships. Like experiment 1a, a minimal meaningful effect size of *f* = 0.4 was selected. An *a priori* power analysis with 95% power indicated 115 participants as the minimal participant size needed. We employed a 2 (erotic stimulation: yes vs. no) × 2 (brand personality: ruggedness vs. sincerity) between-subjects design, and 137 female participants were obtained from Credamo (*M*_age_ = 29.39). Each participant was randomly assigned to one of the four groups and asked to complete a scenario-based survey.

The survey design of Experiment 1b was roughly the same as that of Experiment 1a; the only differences were that participants in Experiment 1b were only women who did not buy products for themselves; Instead they bought a leather bag for their male partners whom they had been dating for at least 6 months and with whom they had relatively stable relationships.

##### Results

We removed two participants from participants who knew the bag brand to avoid interference with the experiment and retained 135 valid participants for further analysis. 86.7% of the participants were in intimate relationships.

The erotic stimulation manipulation test showed that participants in the erotic stimulation group (*M* = 6.22, *SD* = 0.58) rated the sexiness of the pictures significantly higher than those in the scenery group [*M* = 3.96, *SD* = 1.41, *t*(133) = 12.15, *p* = 0.000, η^2^ = 0.53]. Therefore, our manipulation of the erotic stimulation was successful. The brand personality manipulation test revealed that participants in the ruggedness group (*M* = 5.25, *SD* = 1.90) scored significantly higher on the perception of ruggedness than those in the sincerity group [*M* = 2.81, *SD* = 1.75, *t*(133) = 7.78, *p* = 0.000, η^2^ = 0.31]. Thus, our manipulation of brand personality was successful.

**FIGURE 3:**
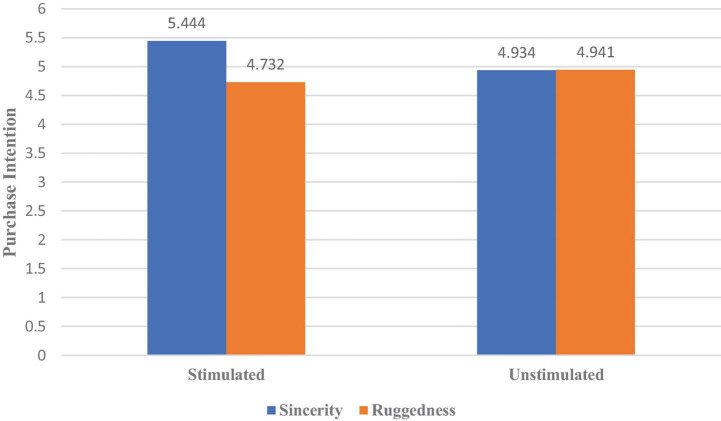
The effect of erotic stimulation on women’s preference for brand personality.

We then conducted an analysis of covariance with erotic stimulation and brand personality as independent variables, purchase intention (Cronbach’s α = 0.794) as a dependent variable, and age and whether they were in a stable relationship as control variables. The results revealed a significant interaction between erotic stimulation and brand personality [η^2^ = 0.084, *F*(1,131) = 14.068, *p* < 0.000] (Figure 3). The simple-effects analysis further showed that erotic stimulation increased women’s preference for the bag embodying sincerity [η^2^ = 0.095, *F*(1,131) = 13.604, *p* < 0.000] but did not affect women’s preference for the bag exhibiting ruggedness [*F*(1,131) = 2.362, *p* = 0.127]. Thus, H2c is supported.

Experiment 1 found that erotic stimulation had different effects on the brand personality preferences of male and female consumers. Specifically, erotic stimulation increases men’s preference for products displaying ruggedness; however, when women in a stable and intimate relationship purchase goods for their partners, erotic stimulation reduces these women’s preference for rugged products but increases their preference for products expressing sincerity.

### Experiment 2: The Effect of Erotic Stimulation on Brand Personality Perception

Experiment 2 explored consumers’ perceptions of brand personality under erotic stimulation. When a woman receives erotic stimulation from other women, she feels that her partner’s possessions are rugged and attractive to other women. Based on strategic intentionality, she will choose sincere products for her partner and expect him to maintain a low profile.

Therefore, Experiment 2 aimed to (1) test the underlying mechanism and verify whether consumers’ perception of brand personality is an unconsciously motivated projection, and (2) extend the external validity by using other experimental stimuli.

#### Experiment 2a: The Effect of Erotic Stimulation on Men’s Perception of Brand Personality

##### Method

Experiment 2a examined how erotic stimulation from women affects men’s evaluation of a product’s brand personality. We employed a double-factor between-subjects design of 2 (erotic stimulation: yes vs. no) × 2 (product owner: male self vs. male colleague) in this study. Like experiment 1, 115 participants are the minimal participant size to detect a medium effect. A total of 153 participants (*M*_age_ = 29.22) were recruited and randomly assigned to one of the four groups. Each participant was given a questionnaire consisting of two separate surveys. In this study, we selected a car as the product that represented personal identity and had social display significance (Birdwell, 1968; Weiss and Johar, 2013).

In the first part, similar to Experiment 1, participants were asked to choose either a spokesperson (in the erotic stimulation group) or a scenery picture (in the non-erotic stimulation group) for an advertisement. In the second part, we manipulated product owners’ perceived personalities and measured consumers’ evaluations of brand personality. In the male self-evaluation group, we asked the participants to imagine that they owned a certain car which they had been driving for a month; In line with the previous study (Weiss and Johar, 2013), we then provided them with the car picture and asked them to answer the question, “How does the car make you feel?” In the control group, the car owner was replaced with a male colleague, but the rest of the descriptions were identical. Then, these participants were asked to evaluate the five dimensions of brand personality (7-point Likert scale) from Aaker’s (1997) framework.

Participants were also asked to answer questions regarding the mate-attraction motive. We used a 7-point Likert scale to measure the strength of participants’ mate-attraction motives, in line with the research by Griskevicius et al. (2007), through the following three questions: “I desire to have a romantic girlfriend,” “I pay great attention to improving my image in women’s eyes,” and “I think it is important to improve my attractiveness to women.” Finally, the participants were asked to answer some questions about themselves, including “How old are you” and “Do you maintain a stable and intimate relationship with your girlfriend?”.

##### Results

The erotic stimulation manipulation test showed that participants in the erotic stimulation group (*M* = 6.37, *SD* = 0.50) rated the sexiness of these pictures significantly higher than those in the scenery group [*M* = 3.49, *SD* = 1.61, t(151) = 14.888, *p* < 0.000, η^2^ = 0.59]. Hence, our manipulation of erotic stimulation was successful.

**FIGURE 4:**
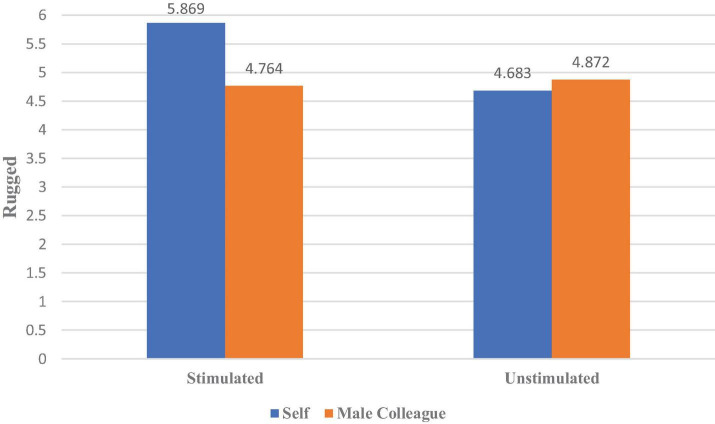
The effect of erotic stimulation on men’s perception of the rugged personality of different owners’ possessions.

We then performed an analysis of covariance with the erotic stimulation and the product owner as independent variables, brand personality as a dependent variable, and age as well as being in a stable relationship or not as control variables. The results (as shown in Figure 4) revealed that the erotic stimulation had main effects on the variable: rugged [η^2^ = 0.108, *F*(1,149) = 24.192, *p* < 0.000], indicating that men tend to think the brand personality of the car is more rugged. The interaction between the erotic stimulation and the product owner significantly influences the variable: rugged [η^2^ = 0.156, *F*(1,149) = 34.946, *p* < 0.000]. There were no main effects or interaction effects on the other brand personalities (*p* > 0.05). The simple-effects analysis further showed that erotic stimulation significantly increased men’s perception of their cars in the rugged dimension [η^2^ = 0.282, *F*(1,149) = 58.168, *p* = 0.000]. However, when men evaluated a male colleague’s car, erotic stimulation had no effect on their perception of ruggedness [*F*(1,149) = 0.223, *p* = 0.486]. Additionally, erotic stimulation had little effect on men’s perception of their cars in the sincerity dimension [*M*_stimulated_ = 5.55, *M*_unstimulated_ = 5.47, *F*(1,149) = 0.286, *p* = 0.775]; hence, H1b was supported.

We established a model with erotic stimulation as the independent variable, mate-attraction motive as the mediating variable, product owner as the moderating variable, ruggedness evaluation as the dependent variable, and current relationship status as the control variable. According to the mediation analysis procedure proposed by Zhao et al. (2017) and the moderated mediation analysis model (Model 15) proposed by Preacher et al. (2007) and Hayes (2017), we conducted a bootstrap test for mediation analysis. In the data analysis, the sample size was set to 5000, and the confidence interval was set to 95%. The results revealed that when the product owner was the participant, the direct effect of the independent variable on the dependent variable, beta, was 0.4894, and the confidence interval was [0.0822, 0.8967], excluding zero. The indirect effect due to the mediating variable, beta, was 0.2701, and the confidence interval was [0.0633, 0.4979], excluding zero. However, when the product owner was a male colleague, the direct effect beta was –0.0733, and the confidence interval was [–0.3587, 0.2121], including zero, and the effect was not significant; the indirect effect beta was 0.0545, and the confidence interval was [–0.0010, 0.1691], including zero, and the effect was not significant. These results indicate that when the product owner was a participant, the effect of erotic stimulation on the product perception of ruggedness was partially mediated by the mate-attraction motive. When the product owner was a male colleague, there was no mediation effect. Thus, the experimental results support the above model.

When men attract potential partners, ruggedness becomes more important, especially because men overestimate its attractiveness to women. The results of Experiment 2a indicate that when a man receives erotic stimulation from women, (1) his mate-attraction motive is aroused; thus, H1a is supported; (2) he perceives that his car’s personality is rugged and attractive to the opposite sex; hence, H1b is supported.

#### Experiment 2b: The Effect of Erotic Stimulation on Women’s Perception of Brand Personality

##### Method

Experiment 2b was designed to examine how erotic stimulation from other women affects women’s evaluations of brand personality in products owned by different holders. We employed a double-factor between-subjects design of 2 (erotic stimulation: yes vs. no) × 2 (product owner: boyfriend vs. male colleague) in this study. A total of 156 participants (*M*_age_ = 29.00) were recruited and randomly assigned to one of the four groups.

Each participant was given a questionnaire consisting of two separate surveys. In the first part, we manipulated erotic stimulation, and the methods were the same as those in Experiment 1a. In the second part, we asked participants to evaluate the brand personality of a car, and the car picture was the same as that in Experiment 2a. In the boyfriend group, we asked the participants to imagine that their male partner owned a car. In the control group, the car owner was described as a male colleague, otherwise identical to Experiment 2a. Participants were asked to rate the car brand personality on a 7-point Likert scale.

Similar to Wang and Griskevicius (2014), we designed three statements about mate-retention motives to measure the mediating variable, including “I need to be careful to protect the intimate relationship with my boyfriend,” “I need to be wary of women my boyfriend meets becoming my potential competitors,” and “I don’t think I need to deliberately consolidate the relationship with my boyfriend.” Participants were asked how much they agreed with the above descriptions on a 7-point Likert scale. Finally, participants were asked to answer questions including “How old are you?” and “Do you have a stable and intimate relationship with your boyfriend?”

##### Results

We first standardized all three items measuring the mate-retention motives and took the factorial scores resulting from the principal component analysis as a composite measure (one single component with an eigenvalue superior to 1 explaining 74.9% of the variance was extracted, Cronbach’s α = 0.795). The erotic stimulation manipulation test revealed that participants in the erotic stimulation group (*M* = 6.20, *SD* = 0.74) rated the sexiness of the pictures significantly higher than those in the scenery group did [*M* = 3.84, *SD* = 1.66, *t*(154) = 11.52, *p* = 0.001, η^2^ = 0.46]. Thus, our manipulation of erotic stimulation was successful.

**FIGURE 5:**
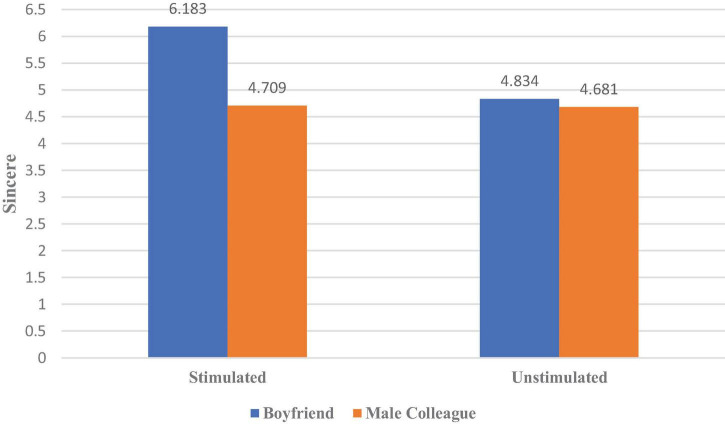
The effect of erotic stimulation on women’s perception of the sincere personality of different owners’ possessions.

Next, we conducted an analysis of covariance with the erotic stimulation and the product owner as independent variables, brand personality as a dependent variable, and age and whether the relationship was stable as control variables. As shown in Figure 5, the results showed that the interaction between the erotic stimulation and the product owner had a remarkable influence on one brand personality: sincere [η^2^ = 0.148, *F*(1,152) = 48.071, *p* < 0.000]. There was no significant interaction effect on other brand personalities (*p* > 0.05). The simple-effects analysis further indicated that the erotic stimulation significantly increased women’s perception of their boyfriends’ cars in the sincere [η^2^ = 0.402, *F*(1,152) = 101.304, *p* < 0.000] dimensions. However, when women evaluated a male colleague’s car, erotic stimulation had no influence on their perceptions of sincere [*F*(1,152) = 0.043, *p* = 0.836] personalities.

We developed a model with erotic stimulation as the independent variable, mate-retention motive as the mediating variable, product owner as the moderating variable, sincerity evaluation as the dependent variable, and current relationship status as the control variable. According to the mediation analysis procedure proposed by Zhao et al. (2017) and the moderated mediation analysis model (Model 15) proposed by Preacher et al. (2007) and Hayes (2017), we performed a bootstrap test for mediation analysis. In the analysis, the sample size was 5000, and the confidence interval was 95%. The results supported that the moderating variable moderated the direct path from the independent variable to the dependent variable. When the product owner was the participant’s boyfriend, the direct effect of the independent variable on the dependent variable, beta, was 0.6039, and the confidence interval was [0.1208, 1.110], excluding zero; the indirect effect beta was 0.3687, and the confidence interval was [0.1516, 0.6633], excluding 0. However, when the product owner was a male colleague, the direct effect beta was 0.0334, and the confidence interval was [–0.2244, 0.2912], including 0, and the effect was not significant; the indirect effect beta was 0.1983, and the confidence interval was [–0.0229, 0.4579], including 0, and the effect was not significant. These results showed that when the product owner was the participant’s boyfriend, the effect of erotic stimulation on the product perception of sincerity was partially mediated by the mate-retention motive; the mediation effect did not take place when the product owner was a male colleague. Hence, the experimental results support the above model.

Sincerity helps men maintain a stable relationship with their partners, responding to women’s preference for a sincere partner. The results of Experiment 2b show that when a woman receives erotic stimulation from other women, (1) her mate-retention motive is stimulated, so H2a is verified; (2) she perceives that her boyfriend’s car’s personality is sincere and conducive to relationship maintenance; hence H2b is supported.

## Discussion

### Summary

Erotic stimulation is widely used in marketing. Previous studies have shown that erotic stimulation from women can actuate men’s mate-attraction motive, prompting them to be more masculine (Baumeister and Vohs, 2004), to pay attention to status-appealing products (Janssens et al., 2011), to prefer buying luxury products (Griskevicius et al., 2007) and conspicuous brands (Sundie et al., 2011). Unlike men, women tend to regard erotic stimulation from other women as a threat to their existing intimate relationships, which in turn arouses their mate-keeping motives (Griskevicius and Kenrick, 2013). From an evolutionary perspective, it is particularly essential for women to maintain relationships with their partners; relationship maintenance can help women get the support of men’s resources, reducing their own investment and risk in childbearing and rearing offspring (Buss and Schmitt, 1993; Wang and Griskevicius, 2014). Previous studies have mainly focused on women’s responses to a potential threat to the romantic relationship (Maner et al., 2007; Wang and Griskevicius, 2014; Krems et al., 2017), while there is little research on how women respond to their partners. This study explores how the mate-retention motive triggered by erotic stimulation from other women is projected onto women’s existing intimate relationships, changing their consumption attitudes and behaviors.

We first investigated the two dimensions (ruggedness and sincerity) of a bag’s brand personality through Experiment 1. The results indicated that erotic stimulation from women increased male consumers’ preference for rugged brand personality. However, female consumers in an intimate relationship, receiving erotic stimulation from other women, prefer a sincere brand personality over a rugged one when shopping for their partners.

Experiment 2 further tested the psychological mechanism behind these behavioral preferences. We found that (1) the erotic stimulation from women arouses men’s mate-attraction motive, causing male participants to make projections associated with the motive onto their possessions, so they perceived their cars as more rugged and attractive to the opposite sex. Similarly, the motive influences projections onto the possessions of others. Specifically, these male participants perceived their male colleagues’ cars as less sincere, suggesting that the perception of brand personality is influenced by perceived motives. And (2) erotic stimulation from other women also affects women. The erotic stimulation arouses women’s mate-retention motive, causing female participants to project the motive onto their partner’s possessions; hence, they perceive their boyfriend’s car as sincerer. However, there was no change in the personality evaluation of male colleagues’ cars by female participants with or without erotic stimulation, showing a motivation-driven bias in the perception of brand personality.

### Contributions

Previous research on erotic stimulation has focused mainly on the effect of heterosexual stimuli on the targeted audience, while our study explores the role of such stimuli on the same-sex audience with a heterosexual orientation. Erotic stimulation marketing targeting men is prevalent in the modern business environment (Reichert et al., 2012). Because sex can attract more attention (Lull and Bushman, 2015), sexual elements in an advertising message are conducive to attracting consumers and establishing a positive connection between the product and the sexy spokesperson (Griskevicius and Kenrick, 2013; King et al., 2015). Although female consumers simultaneously receive sexual stimuli targeted at men, there are few studies on same-sex stimulation.

Erotic stimulation from other women can be perceived as a threat to an existing intimate relationship, triggering women’s mate-keeping motives (Griskevicius and Kenrick, 2013). Driven by such motivation, women’s attitudes and behaviors also change, especially when they are alert to potential threats to their intimate relationships (Wang and Griskevicius, 2014; Li and Meltzer, 2015). Based on these theories, we investigated the impact of same-sex erotic stimulation on women in the marketing field and illustrated the projected effect of the mate-retention motive on brand personality perception and selection. Thus, our study has direct implications for marketing theory and practice.

Furthermore, the research focused on how reproductive motives affect interpersonal relationships and delved into the motivational projection effects generated by erotic stimulation and their mechanisms. People’s perceptions of others in a particular relationship often reflect their desire for interpersonal relationships (Lemay et al., 2007, 2010). The study found that individuals psychologically develop more optimistic perceptions of their partner’s willingness and ability to maintain a relationship when the couple relationship is threatened. This effect can extend from perceptions of people to perceptions of things, producing corresponding projection effects in the consumption domain. The mediating effect of the mate-retention motive and the manipulation of product owners’ projected personality further exemplifies the core mechanism of the relationship motive. Specifically, people only reconstruct their perceptions of their partner’s possessions, but do not change their perceptions of possessions belonging to other opposite-sex persons.

Previous studies have found that the mate-retention motive can stimulate individual coping behavior, including reactions to relationship threats and partners (Buss and Shackelford, 1997). Although many scholars have explored reactions to relationship threats, there is little research on reactions to partners. When an existing relationship is threatened, people directly increase their investment in the relationship (Saad and Gill, 2003; Neal and Lemay, 2014; Sela et al., 2015). Additionally, our study indicated that individuals could adjust their perceptions of the relationship through cognitive reconstruction. It is noteworthy that, in this study, the personality perception of the partner’s possessions only exists in the subjective cognition of the participants, so it can only help individuals gain a sense of relationship security but will not have any substantial effect on the partner or potential threats to the relationship. However, the intrinsic and subtle reactions can further reflect the broad and deep effects of the mate-retention motive. Previous research has paid attention to direct input or confrontational behaviors, while little research has examined how the threat to intimacy affects individuals’ perceptions of their partners. Hence, the cognitive coping strategies explored in this study may advance the research on mate-retention strategies and prompt the academic community to pay more attention to the effects of reproductive motivation.

Additionally, this study showed that the projection effects of such motives are reflected in the perception of brand personality. The abstract concept of a brand has a significant influence on the brand value, customer relations, brand extensions, and cross-cultural marketing, and its core is the mutual reinforcement between brand personality and the self (Fournier, 1998; Aaker et al., 2001; Yorkston et al., 2010). Thus, we regard intimate partners as an essential part of the extended self and innovatively associate brand personality with people’s ideal extended self.

Interestingly, we found that people’s perception of their partner’s personality is not constant but rather a result of construal. The construal reflects the motive of the perceiving subject and is influenced by the situation. Thus, the perception of brand personality comes not only from the brand itself (Simoes et al., 2005), making consumers passively accept the corresponding settings, but also from consumers’ subjective cognitive construction. Specifically, consumers project their motives to the brand, forming a brand’s personality perception. Moreover, the existing research on brand personality perception mainly revolves around the association between consumers’ self and the brand, lacking an investigation into how consumers perceive the association between others and the brand. This study extends the person-brand relationships to intimate relationship contexts and finds that consumers also project their motives on the perception of the association between others and the brand, enriching the understanding of brand personality.

Our findings can help marketers refine their interpretation of consumer psychology and exert the effect of interpersonal motives to achieve better customer experience and brand development. For example, when a man and his female partner attend a car show and plan to buy a car, a sexy female model will stimulate the male consumer’s mate-attraction motive, so a description of the car’s charm may be more appealing to them. However, the female model will inspire the female consumer’s mate-retention motive; hence, a description of the car’s safety and stability may be more effective.

Finally, this study found that there may be conflicts between brand personality traits. For example, an increase in the perception of ruggedness may attenuate the perception of sincerity. Therefore, companies should pay attention to trade-offs and avoid conflicts when investing in brand personality development.

### Directions for Future Research

This study had certain limitations. For example, the activation of the mate-retention motive requires individuals to have an object to protect, while the subjects in this study were unmarried college students. Because we consider college students as consumers, and many studies on marketing and sociology have been conducted with samples of undergraduate college students (Peterson and Merunka, 2014; Wang et al., 2022), we have taken a situational approach to enforcing the stimulus conditions so that future research can explore the impact of same-sex sexual stimuli in the absence of a fixed partner. Due to limited research conditions and the application of situational manipulation, our research samples included single women, but in future studies, we will distinguish between married and unmarried participants to further explore the effects of erotic stimulation on different participants. Then, the research only measured individuals’ perceptions and preferences for the partner’s possessions under motivational projection. Hence, future studies may explore the match between the motivational projection personality and the partner’s actual personality. In addition, the study found that men perceive that their cars are more rugged but less sincere after receiving erotic stimulation, so there is an association or conflict between certain personalities. Thus, future research can explore the association and conflict between brand personality traits.

Finally, relationship motives aroused by erotic stimulation affect perception, and this idea may be extended to the study of other environmental factors. For example, in a disaster, people need to receive care and help from others, which may increase the perception of warmth and friendliness toward others and their possessions.

## Data Availability Statement

The raw data supporting the conclusions of this article will be made available by the authors, without undue reservation.

## Ethics Statement

Ethical review or approval was not required for this study on human participants in accordance with the local legislation and institutional regulations. Written informed consent from the participants was not required to participate in the study in accordance with the national legislation and the institutional regulations.

## Author Contributions

XW developed the theoretical framework and worked on the initial manuscript. XH wrote the initial draft, revised the manuscript, and did further editing on the manuscript. YX was in charge of data collection and analysis. RT supervised the manuscript. All authors contributed to the article and approved the submitted version.

## Conflict of Interest

The authors declare that the research was conducted in the absence of any commercial or financial relationships that could be construed as a potential conflict of interest.

## Publisher’s Note

All claims expressed in this article are solely those of the authors and do not necessarily represent those of their affiliated organizations, or those of the publisher, the editors and the reviewers. Any product that may be evaluated in this article, or claim that may be made by its manufacturer, is not guaranteed or endorsed by the publisher.

## References

[B1] Aaker J. Fournier S. Brasel S. A. (2004). When good brands do bad. *J. Consum. Res.* 31 1–16. 10.1086/383419

[B2] Aaker J. L. (1997). Dimensions of brand personality. *J. Mark. Res.* 34 347–356. 10.2307/3151897

[B3] Aaker J. L. (1999). The malleable self: the role of self-expression in persuasion. *J. Mark. Res.* 36 45–57. 10.2307/3151914

[B4] Aaker J. L. Benet-Martínez V. Garolera J. (2001). Consumption symbols as carriers of culture: a study of Japanese and Spanish brand personality constructs. *J. Pers. Soc. Psychol.* 81 492–508. 10.1037/0022-3514.81.3.492 11554649

[B5] Ahuvia A. C. (2005). Beyond the extended self: loved objects and consumers’ identity narratives. *J. Con. Res.* 32 171–184. 10.1086/429607

[B6] Bakerjr M. D. Jr. Maner J. K. (2008). Risk-taking as a situationally sensitive male mating strategy. *Evol. Hum. Behav.* 29 391–395. 10.1016/j.evolhumbehav.2008.06.001

[B7] Baran S. J. Mok J. J. Land M. Kang T. Y. (1989). You are what you buy: mass-mediated judgments of people’s worth. *J. Commun.* 39 46–54. 10.1111/j.1460-2466.1989.tb01028.x

[B8] Baumeister R. F. Vohs K. D. (2004). Sexual economics: sex as female resource for social exchange in heterosexual interactions. *Pers. Soc. Psychol. Rev.* 8 339–363. 10.1207/s15327957pspr0804_2 15582858

[B9] Belk R. W. (1988). Possessions and the extended self. *J. Con. Res.* 15 139–168. 10.1086/209154

[B10] Berger J. Shiv B. (2011). Food, sex and the hunger for distinction. *J. Con. Psychol.* 21 464–472. 10.1016/j.jcps.2011.01.003

[B11] Birdwell A. E. (1968). A study of the influence of image congruence on consumer choice. *J. Bus.* 41 76–88. 10.3389/fnins.2021.610060 34512233 PMC8427019

[B12] Bleske-Rechek A. Buss D. M. (2006). Sexual strategies pursued and mate attraction tactics deployed. *Pers. Indiv. Diff.* 40 1299–1311. 10.1016/j.paid.2005.11.014

[B13] Borau S. Bonnefon J. F. (2020). Gendered products act as the extended phenotype of human sexual dimorphism: they increase physical attractiveness and desirability. *J. Bus. Res.* 120 498–508. 10.1016/j.jbusres.2019.03.007

[B14] Bradshaw H. K. Rodeheffer C. D. Hill S. E. (2020). Scarcity, sex, and spending: recession cues increase women’s desire for men owning luxury products and men’s desire to buy them. *J. Bus. Res.* 120 561–568. 10.1016/j.jbusres.2019.07.021

[B15] Brough A. R. Wilkie J. E. B. Ma J. Isaac M. S. Gal D. (2016). Is eco-friendly unmanly? the green-feminine stereotype and its effect on sustainable consumption. *J. Consum. Res.* 43 567–582. 10.1093/jcr/ucw044

[B16] Buss D. M. Schmitt D. P. (1993). Sexual strategies theory: an evolutionary perspective on human mating. *Psychol. Rev.* 100 204–232. 10.1037/0033-295x.100.2.204 8483982

[B17] Buss D. M. Shackelford T. K. (1997). From vigilance to violence: mate retention tactics in married couples. *J. Pers. Soc. Psychol.* 72 346–361. 10.1037//0022-3514.72.2.346 9107005

[B18] Cunningham S. J. Turk D. J. Macdonald L. M. Neil Macrae C. N. (2008). Yours or mine? ownership and memory. *Conscious. Cogn.* 17 312–318. 10.1016/j.concog.2007.04.003 17543540

[B19] Dahl D. W. SenGupta J. Vohs K. D. (2009). Sex in advertising: gender differences and the role of relationship commitment. *J. Consum. Res.* 36 215–231. 10.1086/597158

[B20] DeWall C. N. Twenge J. M. Gitter S. A. Baumeister R. F. (2009). It’s the thought that counts: the role of hostile cognition in shaping aggressive responses to social exclusion. *J. Pers. Soc. Psychol.* 96 45–59. 10.1037/a0013196 19210063 PMC2775524

[B21] Fiske A. P. (1992). The four elementary forms of sociality: framework for a unified theory of social relations. *Psychol. Rev.* 99 689–723. 10.1037/0033-295x.99.4.689 1454904

[B22] Fournier S. (1998). Consumers and their brands: developing relationship theory in consumer research. *J. Con. Res.* 24 343–353. 10.1086/209515

[B23] Gerlach T. M. Arslan R. C. Schultze T. Reinhard S. K. Penke L. (2019). Predictive validity and adjustment of ideal partner preferences across the transition into romantic relationships. *J. Pers. Soc. Psychol.* 116 313–330. 10.1037/pspp0000170 28921999

[B24] Griskevicius V. Cialdini R. B. Kenrick D. T. (2006). Peacocks, picasso, and parental investment: the effects of romantic motives on creativity. *J. Pers. Soc. Psychol.* 91 63–76. 10.1037/0022-3514.91.1.63 16834480

[B25] Griskevicius V. Kenrick D. T. (2013). Fundamental motives: how evolutionary needs influence consumer behavior. *J. Con. Psychol.* 23 372–386. 10.1016/j.jcps.2013.03.003

[B26] Griskevicius V. Tybur J. M. Sundie J. M. Cialdini R. B. Miller G. F. Kenrick D. T. (2007). Blatant benevolence and conspicuous consumption: when romantic motives elicit strategic costly signals. *J. Pers. Soc. Psychol.* 93 85–102. 10.1037/0022-3514.93.1.85 17605591

[B27] Gurzki H. Woisetschläger D. M. (2017). Mapping the luxury research landscape: a bibliometric citation analysis. *J. Bus. Res.* 77 147–166. 10.1016/j.jbusres.2016.11.009

[B28] Haselton M. G. Buss D. M. (2000). Error management theory: a new perspective on biases in cross-sex mind reading. *J. Pers. Soc. Psychol.* 78 81–91. 10.1037//0022-3514.78.1.81 10653507

[B29] Hayes A. F. (2017). *Introduction to Mediation, Moderation, and Conditional Process Analysis: A Regression-Based Approach.* New York, NY: Guilford Publications.

[B30] Hill S. E. Durante K. M. (2011). Courtship, competition, and the pursuit of attractiveness: mating goals facilitate health-related risk taking and strategic risk suppression in women. *Pers. Soc. Psychol. Bull.* 37 383–394. 10.1177/0146167210395603 21252381

[B31] Hornik J. Ofir C. Rachamim M. (2017). Advertising appeals, moderators, and impact on persuasion: a quantitative assessment creates a hierarchy of appeals. *J. Advert. Res.* 57 305–318. 10.2501/jar-2017-017

[B32] Janssens K. Pandelaere M. Van den Bergh B. Millet K. Lens I. Roe K. (2011). Can buy me love: mate attraction goals lead to perceptual readiness for status products. *J. Exp. Soc. Psychol.* 47 254–258. 10.1016/j.jesp.2010.08.009

[B33] Johar G. V. SenGupta J. Aaker J. L. (2005). Two roads to updating brand personality impressions: trait versus evaluative inferencing. *J. Mark. Res.* 42 458–469. 10.1509/jmkr.2005.42.4.458 11670861

[B34] Kenrick D. T. Keefe R. C. (1992). Age preferences in mates reflect sex differences in human reproductive strategies. *Behav. Brain Sci.* 15 75–91. 10.1017/s0140525x00067595

[B35] King J. McClelland A. Furnham A. (2015). Sex really does sell: the recall of sexual and non-sexual television advertisements in sexual and non-sexual programmes. *Appl. Cognit. Psychol.* 29 210–216. 10.1002/acp.3095

[B36] Krems J. A. Kenrick D. T. Neel R. (2017). Individual perceptions of self-actualization: what functional motives are linked to fulfilling one’s full potential? *Pers. Soc. Psychol. Bull.* 43 1337–1352. 10.1177/0146167217713191 28903683

[B37] Lambiase J. Reichert T. (2013). “Sex and the marketing of contemporary consumer magazines: how men’s magazines sexualized their covers to compete with Maxim,” in *Sex in Consumer Culture*, (Abingdon: Routledge), 91–110.

[B38] Lemay E. P. Jr. Clark M. S. Feeney B. C. (2007). Projection of responsiveness to needs and the construction of satisfying communal relationships. *J. Pers. Soc. Psychol.* 92 834–853. 10.1037/0022-3514.92.5.834 17484608

[B39] Lemay E. P. Jr. Clark M. S. Greenberg A. (2010). What is beautiful is good because what is beautiful is desired: physical attractiveness stereotyping as projection of interpersonal goals. *Pers. Soc. Psychol. Bull.* 36 339–353. 10.1177/0146167209359700 20179314

[B40] Lemay E. P. Jr. Neal A. M. (2013). The wishful memory of interpersonal responsiveness. *J. Pers. Soc. Psychol.* 104 653–672. 10.1037/a0030422 23088228

[B41] Li N. P. Meltzer A. L. (2015). The validity of sex-differentiated mate preferences: reconciling the seemingly conflicting evidence. *Evol. Behav. Sci.* 9 89–106. 10.1037/ebs0000036

[B42] Li X. Zhang M. (2014). The effects of heightened physiological needs on perception of psychological connectedness. *J. Consum. Res.* 41 1078–1088. 10.1086/678051

[B43] Lull R. B. Bushman B. J. (2015). Do sex and violence sell? a meta-analytic review of the effects of sexual and violent media and ad content on memory, attitudes, and buying intentions. *Psychol. Bull.* 141 1022–1048. 10.1037/bul0000018 26191956

[B44] Lydon J. E. Fitzsimons G. M. Naidoo L. (2003). Devaluation versus enhancement of attractive alternatives: a critical test using the calibration paradigm. *Pers. Soc. Psychol. Bull.* 29 349–359. 10.1177/0146167202250202 15273012

[B45] Malär L. Krohmer H. Hoyer W. D. Nyffenegger B. (2011). Emotional brand attachment and brand personality: the relative importance of the actual and the ideal self. *J. Mark.* 75 35–52. 10.1509/jmkg.75.4.35 11670861

[B46] Maner J. K. Gailliot M. T. Rouby D. A. Miller S. L. (2007). Can’t take my eyes off you: attentional adhesion to mates and rivals. *J. Pers. Soc. Psychol.* 93 389–401. 10.1037/0022-3514.93.3.389 17723055

[B47] Maner J. K. Kenrick D. T. Becker D. V. Robertson T. E. Hofer B. Neuberg S. L. (2005). Functional projection: how fundamental social motives can bias interpersonal perception. *J. Pers. Soc. Psychol.* 88 63–78. 10.1037/0022-3514.88.1.63 15631575

[B48] Maner J. K. Miller S. L. Moss J. H. Leo J. L. Plant E. A. (2012). Motivated social categorization: fundamental motives enhance people’s sensitivity to basic social categories. *J. Pers. Soc. Psychol.* 103 70–83. 10.1037/a0028172 22545747

[B49] Maner J. K. Miller S. L. Rouby D. A. Gailliot M. T. (2009). Intrasexual vigilance: the implicit cognition of romantic rivalry. *J. Pers. Soc. Psychol.* 97 74–87. 10.1037/a0014055 19586241

[B50] Min E. (2012). “When a brand is a sincere friend: compensatory response to social exclusion,” in *Unpublished Doctoral Dissertation*, (Durham: Duke University Press).

[B51] Misra S. Beatty S. E. (1990). Celebrity spokesperson and brand congruence. *J. Bus. Res.* 21 159–173. 10.1016/0148-2963(90)90050-n

[B52] Murray S. L. Holmes J. G. Griffin D. W. (1996). The benefits of positive illusions: idealization and the construction of satisfaction in close relationships. *J. Pers. Soc. Psychol.* 70 79–98. 10.1037/0022-3514.70.1.79

[B53] Neal A. M. Lemay E. P. Jr. (2014). How partners’ temptation leads to their heightened commitment: the interpersonal regulation of infidelity threats. *J. Soc. Personal Relat.* 31 938–957. 10.1177/0265407513512745

[B54] Otterbring T. Ringler C. Sirianni N. J. Gustafsson A. (2018). The abercrombie & fitch effect: the impact of physical dominance on male customers’ status-signaling consumption. *J. Mark. Res.* 55 69–79.

[B55] Otterbring T. Sela Y. (2020). Sexually arousing ads induce sex-specific financial decisions in hungry individuals. *Pers. Individ. Dif.* 152:109576. 10.1016/j.paid.2019.109576

[B56] Peterson R. A. Merunka D. R. (2014). Convenience samples of college students and research reproducibility. *J. Bus. Res.* 67 1035–1041. 10.1016/j.jbusres.2013.08.010

[B57] Plummer J. T. (1985). How personality makes a difference. *J. Advertising Res.* 24 27–31.

[B58] Preacher K. J. Rucker D. D. Hayes A. F. (2007). Addressing moderated mediation hypotheses: theory, methods, and prescriptions. *Multivar. Behav. Res.* 42 185–227. 10.1080/00273170701341316 26821081

[B59] Redlick M. H. Vangelisti A. L. (2018). Affection, deception, and evolution: deceptive affectionate messages as mate retention behaviors. *Evol. Psychol.* 16:1474704917753857. 10.1177/1474704917753857 29433348 PMC10480966

[B60] Reichert T. Carpenter C. (2004). An update on sex in magazine advertising: 1983 to 2003. *Journalism Mass Commun. Q.* 81 823–837. 10.1177/107769900408100407

[B61] Reichert T. Childers C. C. Reid L. N. (2012). How sex in advertising varies by product category: an analysis of three decades of visual sexual imagery in magazine advertising. *J. Curr. Issues Res. Advertising* 33 1–19. 10.1080/10641734.2012.675566

[B62] Reichert T. Heckler S. E. Jackson S. (2001). The effects of sexual social marketing appeals on cognitive processing and persuasion. *J. Advert.* 30 13–27. 10.1080/00913367.2001.10673628

[B63] Reichert T. Lambiase J. (2003). How to get “kissably close”: examining how advertisers appeal to consumers’ sexual needs and desires. *Sex. Cult.* 7 120–136. 10.1007/s12119-003-1006-6

[B64] Reichert T. Lambiase J. Morgan S. Carstarphen M. Zavoina S. (1999). Cheesecake and beefcake: no matter how you slice it, sexual explicitness in advertising continues to increase. *Journalism Mass Commun. Q.* 76 7–20. 10.1177/107769909907600102

[B65] Richard F. D. Bond C. F. Jr. Stokes-Zoota J. J. (2003). One hundred years of social psychology quantitatively described. *Rev. Gen. Psychol.* 7 331–363. 10.1037/1089-2680.7.4.331

[B66] Roux E. Tafani E. Vigneron F. (2017). Values associated with luxury brand consumption and the role of gender. *J. Bus. Res.* 71 102–113. 10.1016/j.jbusres.2016.10.012

[B67] Rusbult C. E. Van Lange P. A. Wildschut T. Yovetich N. A. Verette J. (2000). Perceived superiority in close relationships: why it exists and persists. *J. Pers. Soc. Psychol.* 79 521–545. 10.1037/0022-3514.79.4.521 11045737

[B68] Saad G. (2004). Applying evolutionary psychology in understanding the representation of women in advertisements. *Psychol. Mark.* 21 593–612. 10.1002/mar.20020

[B69] Saad G. Gill T. (2003). An evolutionary psychology perspective on gift giving among young adults. *Psychol. Mark.* 20 765–784. 10.1002/mar.10096

[B70] Samson L. (2018). The effectiveness of using sexual appeals in advertising. *Media Psychol.* 30 184–195. 10.1027/1864-1105/a000194

[B71] Sela Y. Weekes-Shackelford V. A. Shackelford T. K. Pham M. N. (2015). Female copulatory orgasm and male partner’s attractiveness to his partner and other women. *Pers. Individ. Dif.* 79 152–156. 10.1016/j.paid.2015.02.008

[B72] Sengupta J. Dahl D. W. (2008). Gender-related reactions to gratuitous sex appeals in advertising. *J. Con. Psychol.* 18 62–78. 10.1016/j.jcps.2007.10.010

[B73] Septianto F. Seo Y. Errmann A. C. (2021). Distinct effects of pride and gratitude appeals on sustainable luxury brands. *J. Bus. Ethics.* 169 211–224. 10.1007/s10551-020-04484-7

[B74] Simoes C. Dibb S. Fisk R. P. (2005). Managing corporate identity: an internal perspective. *J. Acad. Mark. Sci.* 33 153–168. 10.1177/0092070304268920

[B75] Simpson J. A. Ickes W. Blackstone T. (1995). When the head protects the heart: empathic accuracy in dating relationships. *J. Pers. Soc. Psychol.* 69 629–641. 10.1037/0022-3514.69.4.629

[B76] Solomon M. R. (1983). The role of products as social stimuli: a symbolic interactionism perspective. *J. Con. Res.* 10 319–329. 10.1086/208971

[B77] Sundie J. M. Kenrick D. T. Griskevicius V. Tybur J. M. Vohs K. D. Beal D. J. (2011). Peacocks, porsches, and thorstein veblen: conspicuous consumption as a sexual signaling system. *J. Pers. Soc. Psychol.* 100 664–680. 10.1037/a0021669 21038972

[B78] Swaminathan V. Stilley K. M. Ahluwalia R. (2009). When brand personality matters: the moderating role of attachment styles. *J. Consum. Res.* 35 985–1002. 10.1086/593948

[B79] Vandenbroele J. Van Kerckhove A. Geuens M. (2020). If you work it, flaunt it: conspicuous displays of exercise efforts increase mate value. *J. Bus. Res.* 120 586–598. 10.1016/j.jbusres.2019.01.030

[B80] Wang W. Yi Y. Li J. Sun G. Zhang M. (2022). Lighting up the dark: how the scarcity of childhood resources leads to preferences for bright stimuli. *J. Bus. Res.* 139 1155–1164. 10.1016/j.jbusres.2021.10.058

[B81] Wang Y. Griskevicius V. (2014). Conspicuous consumption, relationships, and rivals: women’s luxury products as signals to other women. *J. Consum. Res.* 40 834–854. 10.1086/673256

[B82] Weiss L. Johar G. V. (2013). Egocentric categorization and product judgment: seeing your traits in what you own (and their opposite in what you don’t). *J. Consum. Res.* 40 185–201. 10.1086/669330

[B83] Willer R. Rogalin C. L. Conlon B. Wojnowicz M. T. (2013). Overdoing gender: a test of the masculine. *Am. J. Sociol.* 118 980–1022. 10.1086/668417

[B84] Yorkston E. A. Nunes J. C. Matta S. (2010). The malleable brand: the role of implicit theories in evaluating brand extensions. *J. Mark.* 74 80–93. 10.1509/jmkg.74.1.80 11670861

[B85] Zhao T. Jin X. Xu W. Zuo X. Cui H. (2017). Mating goals moderate power’s effect on conspicuous consumption among women. *Evol. Psychol.* 15:1474704917723912. 10.1177/1474704917723912 28828887 PMC10481056

