# Supplementary material for: The Influence of Erotic Stimulation on Brand Preference of Male and Female Consumers: From the Perspective of Human Reproductive Motives
Source: Front Psychol. 2022 Jun 7;13:848864. doi: 10.3389/fpsyg.2022.848864 (PMC9210950; doi:10.3389/fpsyg.2022.848864)
Supplement: Supplementary file 1 [file Table_1.docx]

**Appendix 1 The manipulation of the experimental group with erotic stimulation: spokesperson selection for an advertising poster**

A well-known automobile company is shooting a new print advertisement for its new car. Please carefully observe the following four pictures and answer questions. (We make the pictures private due to the copyright issues.)

1. Which model is the most beautiful?

○ A ○ B ○ C ○ D

2. Which model has the best body?

○ A ○ B ○ C ○ D

3. Which model is the most charming?

○ A ○ B ○ C ○ D

4. Which model is the sexiest?

○ A ○ B ○ C ○ D

5. Which model is most attractive to men?

○ A ○ B ○ C ○ D

6. If you were the director of brand marketing, which model would you choose to be the spokesperson of your brand?

○ A ○ B ○ C ○ D

**Appendix 2 The manipulation of the control group without erotic stimulation: selection of an advertising poster**

A well-known travel company is shooting a new print advertisement for its new travel plan. Please carefully observe the following four pictures and answer questions. (We make the pictures private due to the copyright issues)

1. Which poster has the most beautiful view?

○ A ○ B ○ C ○ D

2. Which poster view do you like best?

○ A ○ B ○ C ○ D

3. Which poster style do you like best?

○ A ○ B ○ C ○ D

4. Which poster is the most attractive?

○ A ○ B ○ C ○ D

5. Which poster would you choose as your travel destination?

○ A ○ B ○ C ○ D

6. If you were the director of brand marketing, which poster would you use for the advertising campaign?

○ A ○ B ○ C ○ D
